# Significance of Clinical and Genetic Signatures of Familial Hypercholesterolemia Among Patients With Severe Hypercholesterolemia

**DOI:** 10.1016/j.jacadv.2025.102266

**Published:** 2025-11-03

**Authors:** Hayato Tada, Atsushi Nohara, Soichiro Usui, Kenji Sakata, Masa-aki Kawashiri, Masayuki Takamura

**Affiliations:** a Department of Cardiovascular Medicine, Graduate School of Medical Sciences, Kanazawa University, Kanazawa, Japan; b Department of Clinical Genetics, Ishikawa Prefectural Central Hospital, Kanazawa, Japan; c Department of Internal Medicine, Kaga Medical Center, Kaga, Japan

**Keywords:** familial hypercholesterolemia, genetics, LDL cholesterol, LDL receptor, PCSK9

## Abstract

**Background:**

Familial hypercholesterolemia (FH) is diagnosed based on clinical signs of FH, including family history and/or xanthomas, or pathogenic FH-variant.

**Objectives:**

The authors aimed to clarify whether these signatures are associated with coronary artery disease (CAD) events beyond low density lipoprotein (LDL) cholesterol level among patients with severe hypercholesterolemia.

**Methods:**

We retrospectively reviewed the data of patients with severe hypercholesterolemia (LDL cholesterol ≥180 mg/dL) aged ≥15 years (N = 1,273; male, n = 631) admitted to Kanazawa University Hospital between 2000 and 2022. We divided the patients into 4 groups based on clinical signs of FH and/or presence of FH-variant and assessed the factors associated with CAD events using the Cox proportional hazard model.

**Results:**

We identified 144 CAD events during the 12.4-year median follow-up. Baseline LDL cholesterol level in patients with any signs of FH tended to be significantly higher than non-FH patients. Compared with the reference group of patients without any signs of FH, patients with FH-variant or clinical signs of FH had significantly higher HRs of developing CAD events (HR: 1.44; 95% CI: 1.02-1.86; *P* = 0.007 and HR: 2.27; 95% CI: 1.41-3.13; *P* < 0.001, respectively), whereas those with clinical signs of FH and FH-variant had more than 5-fold higher HR for CAD (HR: 5.02; 95% CI: 2.60-7.44; *P* < 0.001) after adjusting for known risk factors, including LDL cholesterol year score.

**Conclusions:**

Among patients with severe hypercholesterolemia, clinical and genetic signatures of FH are useful for risk discrimination for CAD beyond their hypercholesterolemia.

LDL cholesterol has been the most important causal risk factors for coronary artery disease (CAD).^1^, ^2^, ^3^ In fact, low density lipoprotein (LDL) cholesterol is widely assessed in most of the health checkups, and subjects with severe hypercholesterolemia are typically referred to clinics for further investigations and lipid-lowering therapies. Among them, there are portions of patients with severe hypercholesterolemia whose causes are familial hypercholesterolemia (FH) that is one of the most common inherited monogenic disorders. This disease is caused by genetic mutation(s) in the LDL receptor (*LDLR*) and its associated genes, including apolipoprotein B, proprotein convertase subtilisin/kexin type 9, and LDLR adaptor protein 1.^4^, ^5^, ^6^, ^7^ Globally, several diagnostic criteria for FH are available, including the Dutch Lipid Clinic Network criteria, Simon–Bloom criteria, and Japan Atherosclerosis Society criteria,^8^, ^9^, ^10^ and FH is generally diagnosed based on clinical signs of FH, such as family history or physical findings (cutaneous and/or tendon xanthomas) or the presence of pathogenic FH-variant. It has been suggested that identifying patients with FH among patients with severe hypercholesterolemia is quite important because of their extremely high risk for CAD due to life-long exposure for hypercholesterolemia.^11^^,^^12^ It is true that it has been already shown that a FH-variant confers elevated CAD risk beyond LDL cholesterol levels.^13^ However, whether identifying clinical signs as well as the presence of pathogenic FH-variant of FH would be useful beyond the assessments of LDL cholesterol for the risk stratification of CAD events among patients with severe hypercholesterolemia is unclear. Accordingly, we aimed to assess the clinical impact of these clinical and genetic signatures of FH on the risk of CAD events in patients with severe hypercholesterolemia.

## Patients and methods

### Study population

We analyzed data collected from 2,144 patients with severe hypercholesterolemia (defined as LDL cholesterol ≥180 mg/dL, which is one of the clinical diagnostic criteria of FH in Japan)^10^ who underwent clinical assessment and genetic testing for FH at Kanazawa University Hospital from 2000 to 2022. We excluded patients with missing data (N = 654), those lost to follow-up (N = 387), or those with homozygous FH (N = 6). Finally, we assessed 1,273 patients in this study (Supplemental Figure 1).

### Assessments of clinical signs and pathogenic variant of FH

We assessed: 1) clinical signs of FH, including either of family history of FH or premature CAD (first-degree relatives), or tendon xanthomas or cutaneous xanthomas; and 2) the presence of pathogenic FH-variant, according to clinical criteria of FH 2022 by the Japan Atherosclerosis Society.^10^

### Clinical data assessment

We defined the baseline as the point at which the initial assessments were performed before lipid-lowering therapy was initiated. Hypertension was defined as a systolic blood pressure of ≥140 mm Hg and/or a diastolic blood pressure of ≥90 mm Hg, or the use of any antihypertensive medications. Diabetes was defined based on the diagnostic criteria of Japan Diabetes Society.^8^ We defined smoking status as current smoking. We defined CAD as myocardial infarction, unstable angina, or coronary artery revascularization. Total serum cholesterol, triglyceride, and high density lipoprotein (HDL) cholesterol levels were determined enzymatically using automated instrumentation. The Friedewald formula was used to calculate LDL cholesterol levels when the triglyceride level was <400 mg/dL; otherwise, it was determined enzymatically. LDL cholesterol year score that has been associated with CAD events among patients with FH was calculated as: LDL cholesterol max × (age at diagnosis/statin initiation) + LDL cholesterol at inclusion × (age at inclusion − age at diagnosis/statin initiation).^14^ We documented the lipid values before LDL-lowering therapies were initiated (at baseline), and LDL cholesterol level at follow-up. We assessed variables, including age, sex, hypertension, diabetes, smoking, total cholesterol at baseline, triglyceride at baseline, HDL cholesterol at baseline, LDL cholesterol at baseline, LDL cholesterol at follow-up, LDL cholesterol year score, and prior CAD history as known traditional risk factors for CAD on top of FH category (clinical sign and FH-variant).

### Genetic analysis

A next-generation sequencer was used to sequence the coding regions of the so-called FH genes, including *LDLR*, apolipoprotein B, proprotein convertase subtilisin/kexin type 9, and LDLR adaptor protein 1, as described in a previous study.^15^ The eXome Hidden Markov Model was used to assess the copy number variations at the *LDLR*.^16^ We determined the pathogenicity of the genetic variations using the Standard American College of Medical Genetics and Genomics criteria (“pathogenic” or “likely pathogenic”).^17^ These criteria evaluate multiple standards related to the assessments of pathogenicity, including type of variants, allele frequency, in silico prediction, and functional analyses, and integrates each assessment to determine the final pathogenicity.

### Ethical considerations

The Ethics Committee of Kanazawa University approved this study (2019-321). All procedures met the ethical standards of the Human Research Committee (institutional and national), the 1975 Declaration of Helsinki (revised in 2008), and all other applicable laws and guidelines in Japan. All participants provided informed consent for the genetic analysis.

### Statistical analysis

Continuous variables with normal distribution are presented as mean ± SD, but those without a normal distribution were expressed as medians and IQRs. Data were presented as numbers or percentages. For the independent variables, Student’s *t*-test was used to compare the means of the continuous variables and the nonparametric Wilcoxon Mann-Whitney *U* test was used to compare the median values. Chi-square test or Fisher exact test was used to evaluate categorical variables. The Jonckheere–Terpstra and the Cochran–Armitage trend tests were used for the trends of continuous variables and proportions, respectively. Additionally, a multivariable Cox proportional hazard model was used to identify factors associated with new CAD events, including age, sex, hypertension, diabetes, smoking, LDL cholesterol year score, LDL cholesterol at follow-up, prior CAD history, and FH category (clinical sign and FH-variant). HRs were calculated using the unlikely FH group (group 1) as the reference. Beginning at baseline, cumulative Kaplan-Meier survival curves were generated to compare the times to the first CAD events. We calculated CAD events per 1,000 person-years. Statistical significance was determined with *P* < 0.05. R-4.5.0. software (https://www.r-project.org) was used for all statistical analyses.

## Results

### Clinical characteristics of the participants

**Figure 1:**
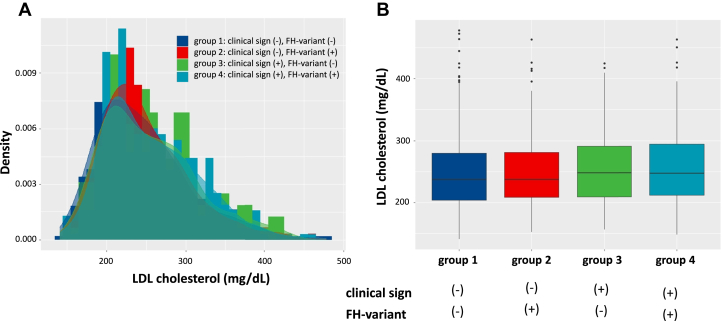
LDL Cholesterol Level According to Clinical/Genetic Stratum of FH (A) Histogram, (B) Boxplot. Group 1 (clinical sign [−], FH-variant [−]): Dark blue. Group 2 (clinical sign [−], FH-variant [+]): Red. Group 3 (clinical sign [+], FH-variant [−]): Green. Group 4 (clinical sign [+], FH-variant [+]): Emerald green FH = familial hypercholesterolemia; LDL = low density lipoprotein.

**Figure 2:**
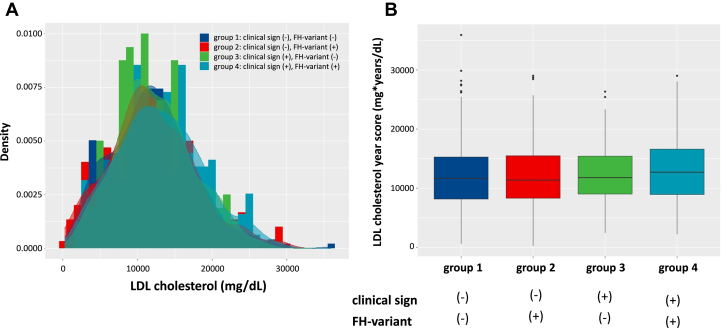
LDL Cholesterol Year Score According to Clinical/Genetic Stratum of FH (A) Histogram, (B) Boxplot. Group 1 (clinical sign [−], FH-variant [−]): Dark blue. Group 2 (clinical sign [−], FH-variant [+]): Red. Group 3 (clinical sign [+], FH-variant [−]): Green. Group 4 (clinical sign [+], FH-variant [+]): Emerald green Abbreviation as in Figure 1.

Table 1 shows the participant’s clinical characteristics. The mean age of the patients was 49 years and 49.6% were men. The median LDL cholesterol level was 241 mg/dL (IQR: 209–286 mg/dL). Moreover, 302 (23.7%) had a history of prior CAD. We found significant trends in variables, such as age, total cholesterol, HDL cholesterol, LDL cholesterol levels, and prior CAD. However, the absolute difference in LDL cholesterol among the groups was minimal, although there was a significant trend (Figure 1). Similar trends were observed in LDL cholesterol year score (Figure 2). Interestingly, we found an opposite trend in LDL cholesterol at follow-up where group 4 who had clinical sign and a FH variant were more intensively treated (Table 1). Pathogenic FH variants identified in this study are presented in Supplemental Table 1.

**Table 1:** Characteristics of the Participants

	All	Group 1: Clinical Sign (−), FH Variant (−)	Group 2: Clinical Sign (−), FH Variant (+)	Group 3: Clinical Sign (+), FH Variant (−)	Group 4: Clinical Sign (+), FH Variant (+)	*P* Value for Trend
	(N = 1,273)	(n = 228)	(n = 298)	(n = 199)	(n = 548)	
Age (y)	49 ± 18	56 ± 14	48 ± 19	49 ± 16	47 ± 18	0.033
Male (%)	631 (49.6%)	114 (50.0%)	147 (49.3%)	98 (49.2%)	272 (49.6%)	0.48
Hypertension (%)	301 (23.6%)	58 (25.4%)	49 (16.4%)	61 (30.7%)	133 (24.3%)	0.34
Diabetes (%)	106 (8.3%)	20 (8.8%)	17 (5.7%)	31 (15.6%)	38 (6.9%)	0.55
Smoking (%)	286 (22.5%)	44 (19.3%)	65 (21.8%)	49 (24.6%)	128 (23.4%)	0.18
Total cholesterol (mg/dL) at baseline	317 [288-359]	314 [284-351]	316 [290-351]	321 [285-368]	322 [289-368]	<0.001
Triglyceride (mg/dL) at baseline	125 [84-173]	125 [80-176]	129 [87-168]	116 [80-160]	130 [85-173]	<0.001
HDL cholesterol (mg/dL) at baseline	47 [40-57]	49 [41-59]	46 [39-56]	46 [40-56]	45 [37-55]	<0.001
LDL cholesterol (mg/dL) at baseline	241 [209-286]	237 [204-280]	237 [209-281]	248 [209-291]	249 [212-294]	<0.001
LDL cholesterol (mg/dL) at follow-up	126 [101-151]	131 [108-154]	128 [106-150]	126 [100-152]	122 [97-147]	<0.001
LDL cholesterol year score (mg∗years/dL)	11,827 [8,519-15,702]	11,639 [8,166-15,296]	11,360 [8,303-15,509]	11,745 [9,023-15,429]	12,716 [8,939-16,607]	<0.001
Prior CAD (%)	302 (23.7%)	25 (11.0%)	48 (16.1%)	51 (25.6%)	178 (32.5%)	<0.001

CAD = coronary artery disease; FH = familial hypercholesterolemia; HDL = high density lipoprotein; LDL = low density lipoprotein.

### Factors associated with CAD events

During the median follow-up period of 12.4 years, these patients had 144 CAD events (Supplemental Table 2). Age (HR: 1.06; 95% CI: 1.02-1.10; *P* < 0.001) (Table 2), male sex (HR: 1.84; 95% CI: 1.04-2.64; *P* < 0.001), hypertension (HR: 2.77; 95% CI: 1.56-3.98; *P* < 0.001), diabetes (HR: 1.54; 95% CI: 1.08-2.00; *P* < 0.001), smoking (HR: 2.53; 95% CI: 1.54-3.52; *P* < 0.001), LDL cholesterol year score (HR: 1.30; 95% CI: 1.10-1.50; *P* < 0.001, per 1,000 mg-year/dL), LDL cholesterol at follow-up (HR: 0.98; 95% CI: 0.97-0.99; *P* < 0.001, per 10 mg), and prior CAD (HR: 3.09; 95% CI: 1.80-5.38; *P* < 0.001) were independently associated with CAD events. Under the circumstances, compared with the reference group of patients without any signs of FH, patients with pathogenic FH-variant or clinical signs of FH had significantly higher HRs of developing CAD events (HR: 1.44; 95% CI: 1.02-1.86; *P* = 0.007 and HR: 2.27; 95% CI: 1.41-3.13; *P* < 0.001, respectively), whereas those with clinical signs of FH and FH-variant had more than 5-fold higher HR for CAD (HR: 5.02; 95% CI: 2.60−7.44; *P* < 0.001) after adjusting for known risk factors, including LDL cholesterol year score and LDL cholesterol at follow-up (Central Illustration). Similar results were obtained in a sensitivity analysis dividing elements of groups (clinical signs of FH and FH-variant), showing that both elements were important (Supplemental Table 3).

**Table 2:** Factors Associated With CAD

	HR	95% CI	*P* Value
Age (per y)	1.06	1.02-1.10	<0.001
Male (yes vs no)	1.84	1.04-2.64	<0.001
Hypertension (yes vs no)	2.77	1.56-3.98	<0.001
Diabetes (yes vs no)	1.54	1.08-2.00	<0.001
Smoking (yes vs no)	2.53	1.54-3.52	<0.001
LDL cholesterol year score (per 1,000 mg∗years/dL)	1.30	1.10-1.50	<0.001
LDL cholesterol (per 10 mg/dL) at follow-up	0.98	0.97-0.99	<0.001
Prior CAD (yes vs no)	3.09	1.80-5.38	<0.001
Group 2: clinical sign (−), FH variant (+) (group 1 as a reference)	1.44	1.02-1.86	0.007
Group 3: clinical sign (+), FH variant (−) (group 1 as a reference)	2.27	1.41-3.13	<0.001
Group 4: clinical sign (+), FH variant (+) (group 1 as a reference)	5.02	2.60-7.44	<0.001

Abbreviations as in Table 1.

### Risk stratification via the diagnostic stratum of FH

**Figure 3:**
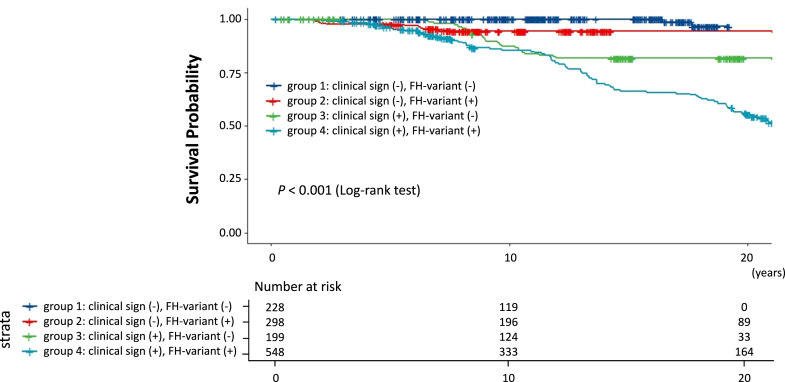
**Kaplan-Meier Survival Curves** Group 1 (clinical sign [−], FH-variant [−]): Dark blue. Group 2 (clinical sign [−], FH-variant [+]): Red. Group 3 (clinical sign [+], FH-variant [−]): Green. Group 4 (clinical sign [+], FH-variant [+]): Emerald green. Abbreviation as in Figure 1.

Compared with the reference group of patients without any signs of FH, patients with pathogenic FH variant and/or clinical signs of FH had a higher risk of developing CAD (Figure 3) (*P* < 0.001). When CAD events were calculated per 1,000 person-years, we found that the event rate was significantly different based on these strata (1.8, 4.4, 6.9, 18.2 per 1,000 person-years, respectively) (Supplemental Figure 2).

**Central Illustration:**
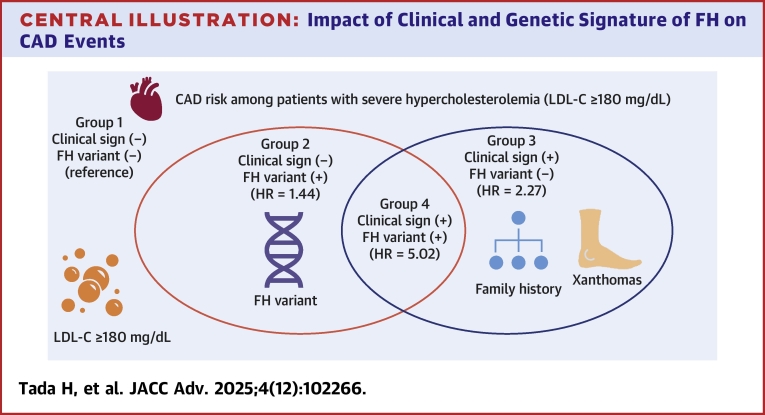
**Impact of Clinical and Genetic Signature of FH on CAD Events** Group 1 (clinical sign [−], FH-variant [−]), Group 2 (clinical sign [−], FH-variant [+]), Group 3 (clinical sign [+], FH-variant [−]), Group 4 (clinical sign [+], FH-variant [+]), HR for CAD events were illustrated according to the group CAD = coronary artery disease; other abbreviation as in Figure 1.

## Discussion

We aimed to clarify whether clinical and genetic signatures of FH were associated with CAD events in patients with severe hypercholesterolemia. We found that 1) clinical risk factors, such as age, sex, hypertension, diabetes, smoking, and LDL cholesterol accumulation were significantly associated with CAD events, 2) clinical and genetic signatures of FH were additively associated with CAD events beyond other clinical risk factors, including LDL cholesterol accumulation.

FH has been associated with an extremely high risk of CAD due to life-long exposure to elevation of LDL cholesterol compared with non-FH. In fact, LDL cholesterol year score calculated in this study was significantly associated with CAD events. However, we also found that clinical and genetic signatures of FH were associated with CAD events beyond LDL cholesterol year score. This fact suggests several important implications. Firstly, LDL cholesterol alone may not be the causal factor of extreme high risk for CAD in patients with FH, although elevation of LDL cholesterol is the primary driver in this matter. One of the potential explanations for this phenomenon would be the inconsistent sensitivity to the exposure to extremely high LDL cholesterol level during the lifespan. In fact, it is more vulnerable in their childhood compared with their adulthood when exposed to the extremely high LDL cholesterol, considering from the fact that the patients with FH who started to treat from their childhood exhibited extremely better prognosis compared with their parents despite under the relatively inadequate LDL cholesterol control.^18^^,^^19^ So, it may be reasonable to see that non-FH patients most of whose LDL cholesterol in their childhood was not so high exhibit better prognosis compared with “true FH patients” who should be exposed to high LDL cholesterol during their childhood. Secondly, clinical and genetic signatures of FH can be used as useful biomarkers for risk stratification of CAD far beyond the assessments of serum LDL cholesterol level. This is a very important notion because these signatures can be used not only for FH diagnosis but also for their risk stratification. In addition, we have already shown that disclosure of FH genetic status in patients with clinical diagnosis of FH led to larger LDL cholesterol reduction in a randomized clinical trial.^20^ Accordingly, determining genetic status of FH on top of clinical diagnosis of FH would further lead to their better prognosis. Thirdly, clinical signs of FH, such as cutaneous and/or tendon xanthomas was significantly associated with CAD risk beyond LDL cholesterol. In this regard, we have previously investigated the factors associated with Achilles tendon thickness among patients with FH and found that it was associated with multiple factors other than LDL cholesterol, such as hypertension and diabetes. In addition, others have shown that Achilles tendon thickness was one of the clinical biomarkers associated with CAD events even among non-FH patients.^21^ Accordingly, it is quite reasonable that clinical sign of FH itself is a useful biomarker for CAD risk on top of LDL cholesterol. In addition, we also found that determining clinical signature of FH on top of FH-variant additively provide useful information for their risk stratification. Based on these results, we strongly suggest assessing these clinical and genetic signatures of FH and then treating them differently based on their risk strata (eg, LDL cholesterol treatment target can be different according to risk strata, although there is currently no clear clinical evidence in this matter). These procedures may be reasonable not only for accurate diagnosis but also for personalized medicine.

### Study Limitations

Our study has several limitations. First, this is a single-center study conducted in a retrospective manner, and the results may not apply to patients referred to others. In addition, small sample size considering the prevalence of severe hypercholesterolemia in the general population may attenuate the results. Second, some patients were excluded because of missing data, which might have affected our results. In fact, the majority of the patients with missing data were due to missing information of LDL cholesterol level at baseline (without treatments). In this regard, 865 patients except for homozygous FH were excluded in the current study due to missing data and/or lost to follow-up. We found that the mean age of them was 36 years old, which was significantly younger than those in the current study. So, it is likely that they have less clinical risk factors, including hypertension, diabetes, LDL cholesterol year score, and clinical signs of FH compared with those in the current study. We assume that excluding them could potentially amplify the degree of associations of variables in the current study, including HRs for CAD events, although we believe that the direction should not be changed. The alternative diagnostic criteria using LDL cholesterol level under treatment, if available, would be needed. Third, we used a short-read next-generation sequencer for genotyping, which may have missed the large copy number variation of *LDLR*. Fourth, we could not fully account for on-treatment LDL cholesterol levels and lipid-lowering therapies in this study, although LDL cholesterol levels at follow-up assessed cross-sectionally were included in out model. Fifth, lack of competing risk modeling limits external validity and causal interpretation in this study.

## Conclusions

Among patients with severe hypercholesterolemia, clinical and genetic signatures of FH are useful for risk discrimination for CAD beyond their hypercholesterolemia.

## Funding support and author disclosures

This work was supported by 10.13039/501100001691JSPS 10.13039/501100001691KAKENHI (22H03330), a grant from the Ministry of Health, Labor and Welfare of Japan (Sciences Research Grant for Research on Rare and Intractable Diseases), the Japanese Circulation Society (Project for Genome Analysis in Cardiovascular Diseases), and the Japan Agency for Medical Research and Development to Dr Tada. The authors have reported that they have no relationships relevant to the contents of this paper to disclose.

## References

[1] Tada H. (2021). Personalized medicine beyond low-density lipoprotein cholesterol to combat residual risk for coronary artery disease. J Atheroscler Thromb.

[2] Ueki Y., Itagaki T., Kuwahara K. (2024). Lipid-lowering therapy and coronary plaque regression. J Atheroscler Thromb.

[3] Okada K., Haze T., Kikuchi S. (2024). Early, intensive and persistent lipid-lowering therapy for secondary prevention of Acute coronary syndrome. J Atheroscler Thromb.

[4] Tada H., Kawashiri M.A., Nohara A., Sekiya T., Watanabe A., Takamura M. (2024). Genetic counseling and genetic testing for familial hypercholesterolemia. Genes (Basel).

[5] Ison H.E., Clarke S.L., Knowles J.W., Adam M.P., Feldman J., Mirzaa G.M., Pagon R.A., Wallace S.E., Amemiya A. (2014). GeneReviews® [Internet].

[6] Takeji Y., Tada H., Takamura M., Tomura A., Harada-Shiba M. (2025). Prevalence and clinical characteristics of familial hypercholesterolemia in patients with acute coronary syndrome according to the Current Japanese guidelines: insight from the EXPLORE-J study. J Atheroscler Thromb.

[7] Michikura M., Ogura M., Matsuki K., Yamaoka M., Makino H., Harada-Shiba M. (2024). Risk assessment for cardiovascular events using achilles Tendon thickness and softness and intima-media thickness in familial hypercholesterolemia. J Atheroscler Thromb.

[8] Austin M.A., Hutter C.M., Zimmern R.L., Humphries S.E. (2004). Genetic causes of monogenic heterozygous familial hypercholesterolemia: a HuGE prevalence review. Am J Epidemiol.

[9] (1991). Risk of fatal coronary heart disease in familial hypercholesterolaemia. Scientific steering committee on behalf of the simon broome register group. BMJ.

[10] Harada-Shiba M., Arai H., Ohmura H. (2023). Guidelines for the diagnosis and treatment of adult familial hypercholesterolemia 2022. J Atheroscler Thromb.

[11] Tada H., Nohara A., Usui S., Sakata K., Kawashiri M.A., Takamura M. (2023). Impact of the severe familial hypercholesterolemia status on atherosclerotic risks. Sci Rep.

[12] Tada H., Nomura A., Nohara A. (2023). Attainment of the low-density lipoprotein cholesterol treatment target and prognosis of heterozygous familial hypercholesterolemia. Atherosclerosis.

[13] Khera A.V., Won H.H., Peloso G.M. (2016). Diagnostic yield and clinical utility of sequencing familial hypercholesterolemia genes in patients with severe hypercholesterolemia. J Am Coll Cardiol.

[14] Tada H., Okada H., Nohara A., Yamagishi M., Takamura M., Kawashiri M.A. (2021). Effect of cumulative exposure to low-density lipoprotein-cholesterol on cardiovascular events in patients with familial hypercholesterolemia. Circ J.

[15] Tada H., Kawashiri M.A., Nomura A. (2018). Oligogenic familial hypercholesterolemia, LDL cholesterol, and coronary artery disease. J Clin Lipidol.

[16] Yamamoto T., Shimojima K., Ondo Y. (2016). Challenges in detecting genomic copy number aberrations using next-generation sequencing data and the eXome Hidden Markov model: a clinical exome-first diagnostic approach. Hum Genome Var.

[17] Richards S., Aziz N., Bale S. (2015). Standards and guidelines for the interpretation of sequence variants: a joint consensus recommendation of the American college of medical genetics and genomics and the Association for molecular pathology. Genet Med.

[18] Luirink I.K., Wiegman A., Kusters D.M. (2019). 20-Year Follow-up of statins in children with familial hypercholesterolemia. N Engl J Med.

[19] Tada H., Kojima N., Yamagami K. (2022). Early diagnosis and treatments in childhood are associated with better prognosis in patients with familial hypercholesterolemia. Am J Prev Cardiol.

[20] Nomura A., Okada H., Nohara A., Kawashiri M.A., Takamura M., Tada H. (2023). Impact of providing genetics-based future cardiovascular risk on LDL-C in patients with familial hypercholesterolemia. J Clin Lipidol.

[21] Matsumoto I., Kurozumi M., Namba T., Takagi Y. (2023). Achilles Tendon thickening as a risk factor of cardiovascular events after percutaneous coronary intervention. J Atheroscler Thromb.

