# Supplementary material for: Significance of Clinical and Genetic Signatures of Familial Hypercholesterolemia Among Patients With Severe Hypercholesterolemia
Source: JACC Adv. 2025 Nov 3;4(12):102266. doi: 10.1016/j.jacadv.2025.102266 (PMC12793841; doi:10.1016/j.jacadv.2025.102266)
Supplement: Supplemental Data [file mmc1.docx]

Supplementary Material

**Supplemental Figure 1. Study flow chart.**

**
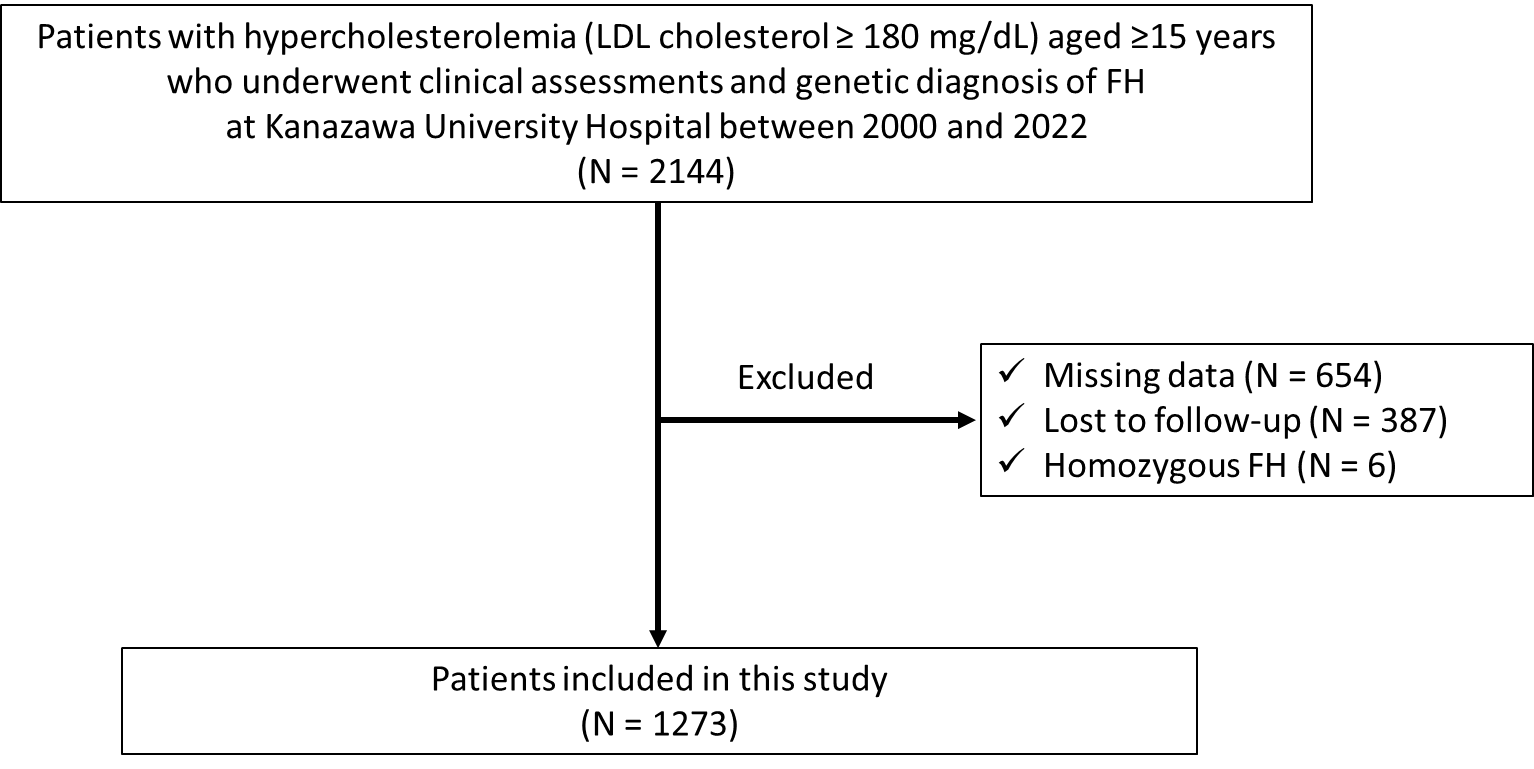
**

**Supplemental Figure 2. CAD event rate based on strata of FH.**

**
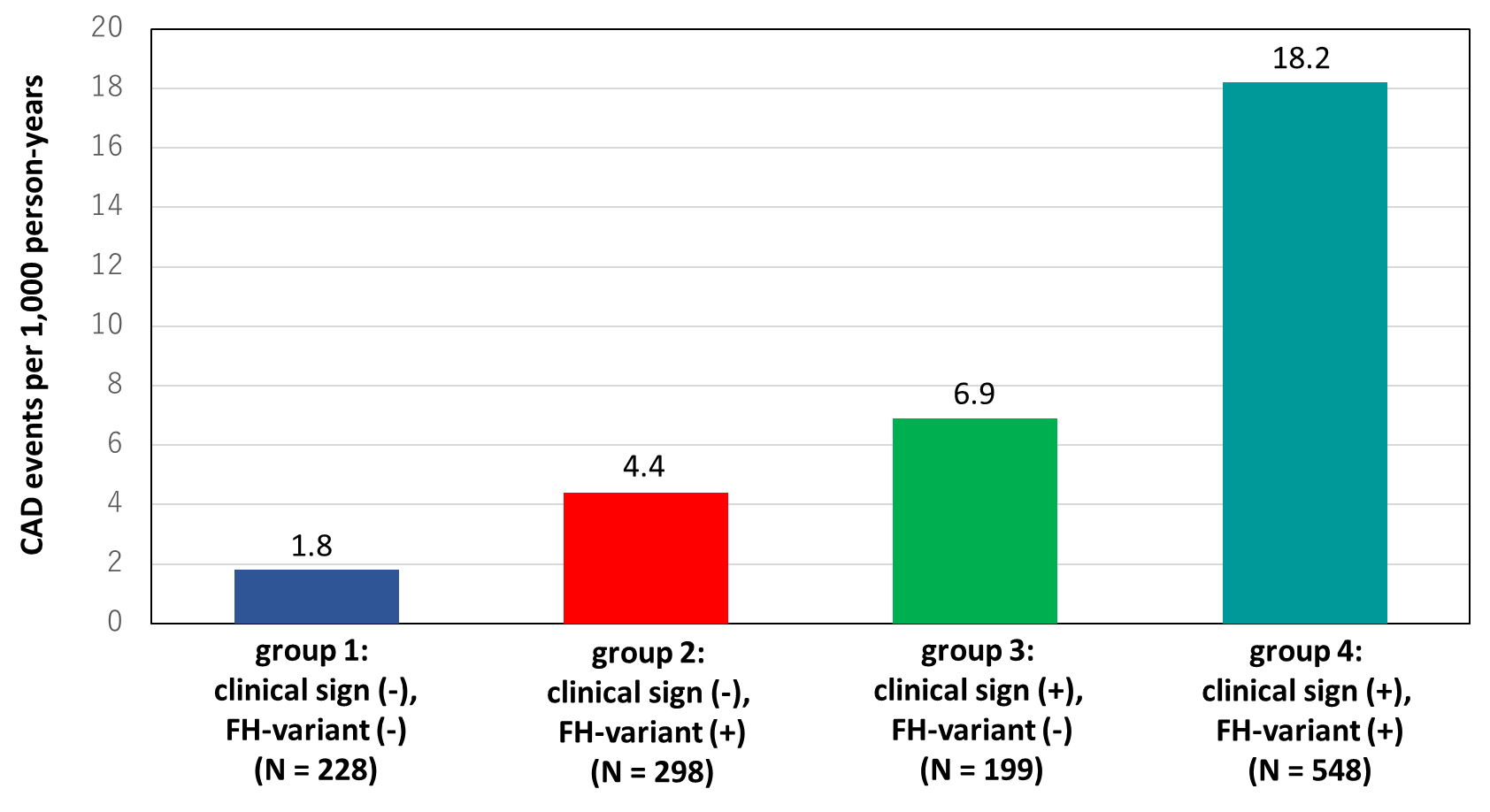
**

**Supplemental Table 1. Identified mutations in FH genes.**

| Gene | Nucleotide change | Mutation Type | Effect on Protein | Number of Patients | ACMG |  |
| --- | --- | --- | --- | --- | --- | --- |
|  |  |  |  |  |  |  |
|  |  |  |  |  |  |  |
| *LDLR* | c.68-1G>C | Splice-cite | NA | 2 | PVS1/PM2/PM4/PP1/PP5 |  |
|  |  |  |  |  | Pathogenic |  |
| *LDLR* | c.130T>G | Missense | p.Trp44Gly | 3 | PM1/PM2/PP3/PP4 |  |
|  |  |  |  |  | Likely pathogenic |  |
| *LDLR* | c.137G>A | Missense | p.Cys46Tyr | 3 | PM1/PM2/PP3/PP5 |  |
|  |  |  |  |  | Likely Pathogenic |  |
| *LDLR* | c.191-2A>G | Splice-cite | NA | 2 | PVS1/PM2/PM4/PP1 |  |
|  |  |  |  |  | Pathogenic |  |
| *LDLR* | c.283T>G | Missense | p.Cys95Gly | 3 | PM1/PM2/PP3/PP4 |  |
|  |  |  |  |  | Likely pathogenic |  |
| *LDLR* | c.313+1G>A | Splice-cite | NA | 3 | PVS1/PM2/PM4 |  |
|  |  |  |  |  | Pathogenic |  |
| *LDLR* | c.361T>G | Missense | p.Cys121Gly | 4 | PM1/PM2/PP3/PP4/PP5 |  |
|  |  |  |  |  | Likely pathogenic |  |
| *LDLR* | c.378del | Frameshift | p.Phe126LeufsTer80 | 2 | PVS1/PM2/PM4/PP1 |  |
|  |  |  |  |  | Pathogenic |  |
| *LDLR* | c.389dupC | Frameshift | p.Asp131ArgfsTer49 | 5 | PVS1/PM1/PM2/PM4/PP5 |  |
|  |  |  |  |  | Pathogenic |  |
| *LDLR* | c.413C>G | Nonsense | p.Ser138Ter | 3 | PVS1/PM2/PM4/PP1/PP5 |  |
|  |  |  |  |  | Pathogenic |  |
| *LDLR* | c.418G>A | Missense | p.Glu140Lys | 4 | PM1/PM2/PP3/PP5 |  |
|  |  |  |  |  | Likely Pathogenic |  |
| *LDLR* | c.478T>C | Missense | p.Cys160Arg | 4 | PM1/PM2/PP3/PP5 |  |
|  |  |  |  |  | Likely Pathogenic |  |
| *LDLR* | c.489G>T | Missense | p.Gln163His | 4 | PM1/PM2/PP3/PP5 |  |
|  |  |  |  |  | Likely Pathogenic |  |
| *LDLR* | c.530C>T | Missense | p.Ser177Leu | 4 | PM1/PM2/PP3/PP5 |  |
|  |  |  |  |  | Likely Pathogenic |  |
| *LDLR* | c.532G>T | Missense | p.Asp178Tyr | 5 | PM1/PM2/PP3/PP5 |  |
|  |  |  |  |  | Likely Pathogenic |  |
| *LDLR* | c.611G>C | Missense | p.Cys204Ser | 6 | PM1/PM2/PP3/PP5 |  |
|  |  |  |  |  | Likely Pathogenic |  |
| *LDLR* | c.642G>C | Missense | p.Trp214Cys | 7 | PM1/PM2/PP3/PP5 |  |
|  |  |  |  |  | Likely Pathogenic |  |
| *LDLR* | c.682G>A | Missense | p.Glu228Gln | 5 | PM1/PM2/PP3/PP5 |  |
|  |  |  |  |  | Likely Pathogenic |  |
| *LDLR* | c.686_689del | Frameshift | p.Glu229AlafsTer35 | 3 | PVS1/PM2/PM4/PP1 |  |
|  |  |  |  |  | Pathogenic |  |
| *LDLR* | c.726G>C | Missense | p.Gln242His | 3 | PM1/PM2/PP3/PP4 |  |
|  |  |  |  |  | Likely Pathogenic |  |
| *LDLR* | c.796G>A | Missense | p.Asp266Asn | 2 | PM1/PM2/PP3/PP4 |  |
|  |  |  |  |  | Likely Pathogenic |  |
| *LDLR* | c.797A>G | Missense | p.Asp266Gly | 5 | PM1/PM2/PM5/PP1/PP3 |  |
|  |  |  |  |  | Likely Pathogenic |  |
| *LDLR* | c.829G>T | Nonsense | p.Glu277Ter | 2 | PVS1/PM2/PM4/PP1 |  |
|  |  |  |  |  | Pathogenic |  |
| *LDLR* | c.874delC | Frameshift | p.Leu292TrpfsTer78 | 3 | PVS1/PM2/PM4/PP1 |  |
|  |  |  |  |  | Pathogenic |  |
| *LDLR* | c.901G>T | Missense | p.Asp301Tyr | 4 | PM1/PM2/PP3/PP5 |  |
|  |  |  |  |  | Likely Pathogenic |  |
| *LDLR* | c.902A>T | Missense | p.Asp301Val | 4 | PM1/PM2/PP3/PP5 |  |
|  |  |  |  |  | Likely Pathogenic |  |
| *LDLR* | c.937T>G | Missense | p.Cys313Gly | 3 | PM1/PM2/PP3/PP4 |  |
|  |  |  |  |  | Likely Pathogenic |  |
| *LDLR* | c.939C>A | Missense | p.Cys313Ter | 3 | PVS1/PM2/PM4/PP5 |  |
|  |  |  |  |  | Pathogenic |  |
| *LDLR* | c.940+2T>C | Splice-cite | NA | 3 | PVS1/PM2/PM4/PP1 |  |
|  |  |  |  |  | Pathogenic |  |
| *LDLR* | c.967G>A | Missense | p.Gly323Ser | 4 | PM1/PM2/PP3/PP4 |  |
|  |  |  |  |  | Likely Pathogenic |  |
| *LDLR* | c.1007_1010delACGA | Frameshift | p.Tyr336CysfsTer33 | 7 | PVS1/PM2/PM4/PP5 |  |
|  |  |  |  |  | Pathogenic |  |
| *LDLR* | c.1012T>A | Missense | p.Cys338Ser | 2 | PM1/PM2/PP3/PP5 |  |
|  |  |  |  |  | Likely Pathogenic |  |
| *LDLR* | c.1056C>A | Nonsense | p.Cys352Ter | 2 | PVS1/PM2/PM4/PP5 |  |
|  |  |  |  |  | Pathogenic |  |
| *LDLR* | c.1062dupT | Frameshift | p.Ile355TyrfsTer3 | 2 | PVS1/PM2/PM4/PP5 |  |
|  |  |  |  |  | Pathogenic |  |
| *LDLR* | c.1067A>T | Missense | p.Asp356Val | 2 | PM1/PM2/PP3/PP4 |  |
|  |  |  |  |  | Likely Pathogenic |  |
| *LDLR* | c.1069G>T | Nonsense | p.Glu357Ter | 2 | PVS1/PM2/PM4 |  |
|  |  |  |  |  | Pathogenic |  |
| *LDLR* | c.1114_1115insC | Frameshift | p.Glu372AlafsTer9 | 5 | PVS1/PM2/PM4/PP5 |  |
|  |  |  |  |  | Pathogenic |  |
| *LDLR* | c.1187-2A>G | Splice-cite | NA | 3 | PVS1/PM2/PM4/PP5 |  |
|  |  |  |  |  | Pathogenic |  |
| *LDLR* | c.1207T>C | Missense | p.Phe403Leu | 3 | PM1/PM2/PP3/PP5 |  |
|  |  |  |  |  | Likely Pathogenic |  |
| *LDLR* | c.1245_1249dupCCGGA | Frameshift | p.Ser417ThrfsTer12 | 4 | PVS1/PM2/PM4/PP4 |  |
|  |  |  |  |  | Pathogenic |  |
| *LDLR* | c.1246C>T | Missense | p.Arg416Trp | 4 | PM1/PM2/PP3/PP4 |  |
|  |  |  |  |  | Likely Pathogenic |  |
| *LDLR* | c.1252G>A | Missense | p.Glu418Lys | 4 | PM1/PM2/PP3/PP4 |  |
|  |  |  |  |  | Likely Pathogenic |  |
| *LDLR* | c.1285G>A | Missense | p.Val429Leu | 4 | PM1/PM2/PP3/PP4 |  |
|  |  |  |  |  | Likely Pathogenic |  |
| *LDLR* | c.1297G>C | Missense | p.Asp433His | 4 | PM1/PM2/PP3/PP5 |  |
|  |  |  |  |  | Likely Pathogenic |  |
| *LDLR* | c.1328G>C | Missense | p.Trp443Ser | 5 | PM1/PM2/PP3/PP4 |  |
|  |  |  |  |  | Likely Pathogenic |  |
| *LDLR* | c.1339T>C | Missense | p.Ser447Pro | 4 | PM1/PM2/PP3/PP5 |  |
|  |  |  |  |  | Likely Pathogenic |  |
| *LDLR* | c.1340C>G | Missense | p.Ser447Cys | 3 | PM1/PM2/PP3/PP5 |  |
|  |  |  |  |  | Likely Pathogenic |  |
| *LDLR* | c.1432G>A | Missense | p.Gly478Arg | 4 | PM1/PM2/PP3/PP4 |  |
|  |  |  |  |  | Likely Pathogenic |  |
| *LDLR* | c.1466A>G | Missense | p.Tyr489Cys | 8 | PM1/PM2/PP3/PP5 |  |
|  |  |  |  |  | Likely Pathogenic |  |
| *LDLR* | c.1474G>A | Missense | p.Asp492Asn | 4 | PM1/PM2/PP3/PP4 |  |
|  |  |  |  |  | Likely Pathogenic |  |
| *LDLR* | c.1502C>T | Missense | p.Ala501Val | 5 | PM1/PM2/PP3/PP4 |  |
|  |  |  |  |  | Likely Pathogenic |  |
| *LDLR* | c.1567G>A | Missense | p.Val523Met | 3 | PM1/PM2/PP3/PP5 |  |
|  |  |  |  |  | Likely Pathogenic |  |
| *LDLR* | c.1586+1G>A | Splice-cite | NA | 2 | PVS1/PM2/PM4/PP4 |  |
|  |  |  |  |  | Pathogenic |  |
| *LDLR* | c.1652_1662delACATCTACTCG | Frameshift | p.Asp551AlafsTer4 | 5 | PVS1/PM2/PM4/PP4 |  |
|  |  |  |  |  | Pathogenic |  |
| *LDLR* | c.1702C>G | Missense | p.Leu568Val | 16 | PM1/PM2/PP3/PP5 |  |
|  |  |  |  |  | Likely Pathogenic |  |
| *LDLR* | c.1705+1G>C | Splice-cite | NA | 6 | PVS1/PM2/PM4/PP4 |  |
|  |  |  |  |  | Pathogenic |  |
| *LDLR* | c.1706A>G | Missense | p.Asp569Gly | 2 | PM1/PM2/PP3/PP4 |  |
|  |  |  |  |  | Likely Pathogenic |  |
| *LDLR* | c.1727A>G | Missense | p.Tyr576Cys | 5 | PM1/PM2/PP3/PP4 |  |
|  |  |  |  |  | Likely Pathogenic |  |
| *LDLR* | c.1731G>T | Missense | p.Trp577Cys | 5 | PM1/PM2/PP3/PP4 |  |
|  |  |  |  |  | Likely Pathogenic |  |
| *LDLR* | c.1778dupG | Frameshift | p.Asn594GlnfsTer9 | 5 | PVS1/PM2/PM4/PP4 |  |
|  |  |  |  |  | Pathogenic |  |
| *LDLR* | c.1783C>T | Missense | p.Arg595Trp | 4 | PM1/PM2/PP3/PP4 |  |
|  |  |  |  |  | Likely Pathogenic |  |
| *LDLR* | c.1845+2T>C | Splice-cite | NA | 4 | PVS1/PM2/PM4/PP4 |  |
|  |  |  |  |  | Pathogenic |  |
| *LDLR* | c.1859G>C | Missense | p.Trp620Ser | 4 | PM1/PM2/PP3/PP4 |  |
|  |  |  |  |  | Likely Pathogenic |  |
| *LDLR* | c.1868T>A | Missense | p.Ile623Asn | 3 | PM1/PM2/PP3/PP4 |  |
|  |  |  |  |  | Likely Pathogenic |  |
| *LDLR* | c.1897C>T | Missense | p.Arg633Cys | 4 | PM1/PM2/PP3/PP4 |  |
|  |  |  |  |  | Likely Pathogenic |  |
| *LDLR* | c.1925T>C | Missense | p.Leu642Ser | 3 | PM1/PM2/PP3/PP4/PP5 |  |
|  |  |  |  |  | Likely Pathogenic |  |
| *LDLR* | c.1998G>C | Missense | p.Trp666Cys | 4 | PM1/PM2/PP3/PP4 |  |
|  |  |  |  |  | Likely Pathogenic |  |
| *LDLR* | c.2050G>T | Missense | Ala684Ser | 4 | PM1/PM2/PP3/PP4 |  |
|  |  |  |  |  | Likely Pathogenic |  |
| *LDLR* | c.2054C>T | Missense | p.Pro685Leu | 26 | PM1/PM2/PP3/PP4/PP5 |  |
|  |  |  |  |  | Likely Pathogenic |  |
| *LDLR* | c.2096C>T | Missense | p.Pro699Leu | 4 | PM1/PM2/PP3/PP4 |  |
|  |  |  |  |  | Likely Pathogenic |  |
| *LDLR* | c.2389G>A | Missense | p.Val797Met | 6 | PM1/PM2/PP3/PP4 |  |
|  |  |  |  |  | Likely Pathogenic |  |
| *LDLR* | c.2390-4_2393delACAGTGCT | Splice-cite | NA | 2 | PVS1/PM2/PM4/PP4 |  |
|  |  |  |  |  | Pathogenic |  |
| *LDLR* | c.2416delG | Frameshift | p.Val806SerfsTer123 | 3 | PVS1/PM2/PM4/PP4 |  |
|  |  |  |  |  | Pathogenic |  |
| *LDLR* | c.2416dupG | Frameshift | p.Val806GlyfsTer11 | 4 | PVS1/PM2/PM4/PP4 |  |
|  |  |  |  |  | Pathogenic |  |
| *LDLR* | c.2431A>T | Nonsense | p.Lys811Ter | 119 | PVS1/PM2/PM4/PP4 |  |
|  |  |  |  |  | Pathogenic |  |
| *LDLR* | c.2500G>A | Missense | p.Asp834Asn | 4 | PVS1/PM2/PM4/PP4 |  |
|  |  |  |  |  | Pathogenic |  |
| *LDLR* | c.2579C>T | Missense | p.Ala860Val | 5 | PVS1/PM2/PM4/PP4 |  |
|  |  |  |  |  | Pathogenic |  |
| *LDLR* | c.313-?_2311+?del | Large deletion | Truncated protein | 2 | PVS1/PM2/PM4/PP4 |  |
|  |  |  |  |  | Pathogenic |  |
| *LDLR* | c.1186-?_1587+?dup | Large duplication | Truncated protein | 2 | PVS1/PM2/PM4/PP4 |  |
|  |  |  |  |  | Pathogenic |  |
| *LDLR* | c.1845-?_2141+?del | Large deletion | Truncated protein | 1 | PVS1/PM2/PM4/PP4 |  |
|  |  |  |  |  | Pathogenic |  |
| *LDLR* | c.2141-?_2311+?del | Large deletion | Truncated protein | 3 | PVS1/PM2/PM4/PP4 |  |
|  |  |  |  |  | Pathogenic |  |
| *PCSK9* | c.94G>A | Missense | p.Glu32Lys | 30 | PS1/PS3/PP3/PP4/PP5 |  |
|  |  |  |  |  | Pathogenic |  |

ACMG, American College of Medical Genetics; *LDLR*, low-density lipoprotein receptor; *PCSK9*, proprotein convertase subtilisin/kexin type 9; FH, familial hypercholesterolemia.

**Supplemental Table 2. CAD events during the follow-up period**

| Type of CAD | All (N = 1273) |
| --- | --- |
| Myocardial infarction | 28 (2.2 %) |
| Unstable angina | 49 (3.8 %) |
| Coronary artery revascularization | 67 (5.3 %) |
| Total | 144 (11.3 %) |

**Supplemental Table 3. Factors associated with CAD**

| Variable | HR | 95% CI | *P*-value |
| --- | --- | --- | --- |
| Age (per year) | 1.05 | 1.03 – 1.07 | < 0.001 |
| Male (yes vs. no) | 1.98 | 1.12 – 2.84 | < 0.001 |
| Hypertension (yes vs. no) | 2.92 | 1.88 – 3.96 | < 0.001 |
| Diabetes (yes vs. no) | 1.61 | 1.11 – 2.11 | < 0.001 |
| Smoking (yes vs. no) | 2.88 | 1.72 – 4.04 | < 0.001 |
| LDL cholesterol year score (per 1,000 mg*years/dL) | 1.31 | 1.11 – 1.51 | < 0.001 |
| LDL cholesterol (per 10 mg/dL) at follow-up | 0.98 | 0.97 – 0.99 | < 0.001 |
| prior CAD (yes vs. no) | 3.24 | 1.90 – 4.58 | < 0.001 |
| clinical sign (+) | 2.68 | 1.44 – 3.92 | < 0.001 |
| FH-variant (+) | 1.76 | 1.08 – 2.44 | < 0.001 |
